# Detection of Authenticity and Quality of the Turkish Delights (Lokum) by Means of Conventional and Fast Field Cycling Nuclear Magnetic Resonance Relaxometry

**DOI:** 10.1021/acs.jafc.1c00943

**Published:** 2021-06-21

**Authors:** Pelin Pocan, Magdalena Knapkiewicz, Adam Rachocki, Mecit Halil Oztop

**Affiliations:** † Department of Food Engineering, Middle East Technical University, 06800 Ankara, Turkey; ‡ Institute of Molecular Physics, Polish Academy of Sciences, 60-179 Poznań, Poland

**Keywords:** fast field cycling (FFC) NMR relaxometry, time domain (TD) NMR relaxometry, water dynamics, soft candies, food gels

## Abstract

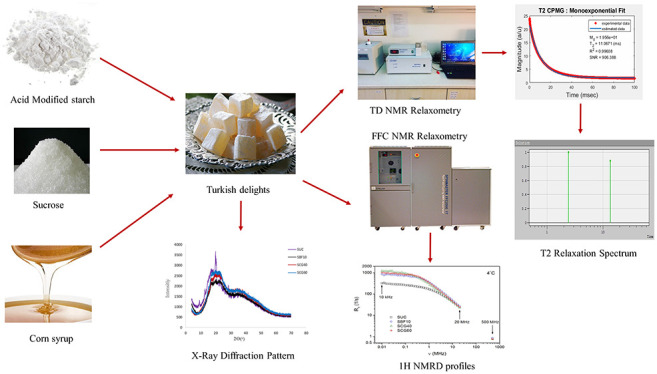

Turkish delights (lokum) are traditional confectionery products that contain mainly sucrose as the sugar source and starch as the gelling agent. However, manufacturers sometimes might prefer to use corn syrup instead of sucrose to decrease the cost. This jeopardizes the originality of Turkish delights and leads to production of adulterated samples. In this study, Turkish delights were formulated using sucrose (original sample) and different types of corn syrups (SBF10, SCG40, and SCG60). Results clearly indicated that corn-syrup-containing samples had improved textural properties and were less prone to crystallization. However, this case affected authenticity of the products negatively. Both time domain nuclear magnetic resonance (TD NMR) and fast field cycling nuclear magnetic resonance (FFC NMR) techniques were found to be effective to discriminate the original samples from the corn-syrup-containing samples. In addition, quantitative analysis of FFC NMR showed that, apart from the rotational motions, molecules in Turkish delights (mainly water and also sugar molecules) undergo two types of translational dynamics.

## 1 Introduction

Soft candy products are the perfect examples for the composite gel systems, which are composed of high amounts of sugar together with different types of gelling agents, such as starch, gelatin, or pectin.^1^ Turkish delights (lokum) are also an example of these soft candy products and known as traditional sugar-based jelly confections, which contain starch as the gelling agent.^2^ In accordance with Turkish food legislation, Turkish delight (lokum) is prepared using sucrose, starch, drinking water, citric acid, or tartaric acid as the main ingredients.^3^ As its name implies, it is a well-known traditional confectionery product especially in Turkey, but it is also popular in Greece, Middle Eastern countries, and the Balkans.^4^ Turkish delights are also very important confectionery products in terms of their economic value and market share in Turkey. Therefore, they are protected under Turkish legislation covering ingredients and production methods. Therefore, it is vital to determine the originality and authenticity of the production of Turkish delights, especially in terms of the ingredients. According to the national legislations, the only sugar type that can be used to produce lokum is defined as white sugar, which is sucrose.^3^ However, confectionery manufacturers prefer to use corn syrup instead of sucrose for various purposes, such as crystallization inhibition.^5^ As known from the previous studies, corn syrup can be used as a crystallization inhibitor to improve the shelf life of the confectionery products.^6^ It was also stated that, as the amount of corn syrup increased in the formulations, the smaller crystals were obtained, leading to the formation of more desirable confectionery products in terms of both textural and sensorial properties.^7^ In addition, manufacturers might prefer corn syrups to decrease the cost of ingredients. However, utilization of corn syrups [especially high-fructose corn syrup (HFCS)] as a sweetener is a controversial issue because they can cause several health concerns. For example, in previous studies, it was stated that consumption of HFCS, which is more lipogenic than sucrose, might increase the risk for non-alcoholic fatty liver disease (NAFLD) and dyslipidemia.^8^ In addition to these health concerns about corn syrups, utilization of them especially for the production of Turkish delights is also controversial because it directly affects the authenticity of the products protected by legislations as mentioned before, and Turkish delights produced using corn syrup can even be considered as “adulterated”.

To understand the type of sugar and discriminate the adulterated samples from the original samples, time domain nuclear magnetic resonance (TD NMR) can be used as a promising tool. As a result of its non-destructive, time-saving, and less laborious nature, it could be considered as an important characterization technique to detect the quality of the food products.^9^ As well as quality detection, TD NMR is also an important tool to determine the originality and adulteration in food samples. For instance, it was used in several studies to detect the adulteration in various food products, such as milk,^10^ olive oil,^11^ frankfurter,^12^ and wine and fruit juices.^13^ During TD NMR experiments, *T*_1_ (spin–lattice) and *T*_2_ (spin–spin) relaxation times of the samples was measured using different pulse sequences.^14^*T*_1_ times were generally associated with crystal structures found in samples,^15^ while *T*_2_ times can be used to understand polymer–polymer and polymer–water interactions in gel systems,^16^ gelation behavior of different types of proteins,^17^ and emulsification and hydration behavior of various food systems.^18^ As a result of the multi-compartment nature of gel systems, multi-exponential analysis of relaxation decays is a more useful approach to obtain information about the different proton pools that exist in gel matrices, and these proton pools can be used as a fingerprint to analyze the quality and microstructure of the food gels.^19^ This multi-exponential approach was used in various studies in which low-field TD NMR was used to characterize the different types of confectionery gels, such as d-allulose-containing gelatin,^1^ starch,^20^ and pectin^21^ based soft candies, and in all of these studies, existence of different proton pools with different *T*_2_ relaxation times was emphasized. These proton pools were used as a fingerprint and quality detection method for each soft candy product. In these mentioned studies, conventional NMR methods were used, leading to measurement of *T*_1_ and *T*_2_ relaxation times at a single magnetic field (at 20.34 MHz resonance frequency). Although TD NMR enables researchers to obtain various information from different proton pools, detailed analysis about the water dynamics in gel matrices could not be obtained using a single resonance frequency system.

The importance of fast field cycling nuclear magnetic resonance (FFC NMR) is revealed at this point. FFC NMR relaxometry is the preferred technique for obtaining the frequency (or magnetic field) dependence of proton spin–lattice relaxation rates (*R*_1_). It is also referred to as nuclear magnetic relaxation dispersion (NMRD).^22^ Thanks to FFC technology, it is the only low-field NMR technique that detects the *T*_1_ (spin–lattice) relaxation time as a function of the magnetic field strength over a wide range of frequencies (from a few kilohertz to tens of megahertz),^23^ enabling researchers to obtain detailed analysis of molecular dynamics in a single experiment and to understand the mechanism of motion,^22^ such as dimensionality of translation diffusion.^24^ As a result of its unique potential to describe water dynamics, it is suitable to characterize gel systems, such as predicting the effect of gelation on diffusion in renewable ionic gels^25^ and understanding the solvent dynamics within supramolecular gels via *T*_1_ relaxation times and diffusion coefficient^26^ and mechanism of water dynamics in hyaluronic dermal fillers.^24^ In addition to its potential to characterize the gel systems, it is also suitable to be used as a quality control tool, such as detecting the shelf life of fruits^27^ and milk products.^28^ In previous studies, it was also used to detect the geographical origins of vinegars,^29^ to obtain diffusion coefficients of vegetable oils,^30^ and to characterize wine^23^ and honey.^31^ In a recent review by Ates et al., applications of FFC NMR have been discussed for different food categories, including dairy products, confectionaries (chocolate and gelatin candies), meat, honey, oil, and fruits.^32^ However, to the best of our knowledge, there is no study in the literature that examines the application of FFC NMR relaxometry on Turkish delights (lokum) to detect the quality and authenticity of the samples. In the present work, the FFC NMR relaxometry technique will be used to try to discriminate the original Turkish delight samples (sucrose-containing samples) from the corn-syrup-containing (adulterated) samples. FFC NMR relaxometry was also used as not only an authenticity tool but also a quality detection tool. Because different types of sugar sources directly affect the water mobility in gel matrices of Turkish delights, quantitative analysis of NMR relaxometry data was performed with well-defined parameters to explain the water dynamics of the samples, which also directly affects their quality properties, such as their texture, color, etc. In addition to the FFC NMR relaxometry technique, the TD NMR technique was also used to characterize Turkish delight samples. The main objective of this study is to reveal the potential of both TD NMR and FFC NMR techniques to detect the authenticity and quality of Turkish delights.

## 2 Materials and Methods

### 2.1 Materials

Sucrose (Bal Küpü, Aksaray, Turkey) was purchased from a local market in Ankara, Turkey. Corn syrups with commercial names SBF10, SCG40, and SCG60 were kindly provided by Sunar Mısır A.Ş. (Adana, Turkey). The total soluble solid content (Brix) and glucose or glucose/fructose content of these corn syrups were given in Table 1. Acid-modified starch was kindly provided by Kervan Gıda A.Ş. (Istanbul, Turkey). Citric acid monohydrate was purchased from Sigma-Aldrich Chemical Co. (St. Louis, MO, U.S.A.). Distilled water was used in all formulations.

**Table 1:** Specifications of Corn Syrup Types That Were Used in the Production of Turkish Delights

corn syrup name	Brix (°Bx)	glucose (%)	fructose (%)
SBF10 (glucose/fructose syrup)	79	36	10
SCG40 (glucose syrup)	83	40	
SCG60 (glucose syrup)	82	60	

### 2.2 Methods

#### 2.2.1 Preparation of the Samples

Turkish delights were prepared according to the method of Ilhan et al.,^20^ with some modifications.

A total of 10 g of starch was mixed with 2 times the amount of water (20 g) by its weight and gelatinized in an oil bath at 140 °C for 5 min until it was dissolved completely. During this time, 54 g of sugar (sucrose or corn syrup) and 16 mL of water boiled up to 115 °C before mixed with starch water. A total of 0.1 g of citric acid was also added to this sugar mixture for all formulations. Cooking was continued at 125 °C in an oil bath. Afterward, the mixture was poured into starch molds with dimensions of 2.5 × 2.5 × 2 cm and kept at room temperature (25 °C) for 48 h. Composition (%, w/w) of the Turkish delights was given in Table 2. Original Turkish delight samples (SUC) were prepared using only powder sugar (sucrose), while other samples were prepared using different type of corn syrups as the sugar source. They were classified with the same name as the corn syrups that they contain (SBF10, SCG40, and SCG60).

**Table 2:** Turkish Delights Formulated with Different Types of Sugar (Corn Syrup or Sucrose) (%, w/w)

sample name	starch (%)	sucrose (%)	corn syrup (%)	citric acid (%)
SUC	10	54		0.1
SBF10	10		54	0.1
SCG40	10		54	0.1
SCG60	10		54	0.1

#### 2.2.2 Moisture Content Determination

The moisture content of the different formulations was measured at 70 °C for 4 h in a vacuum oven (DAIHAN, Germany). Weight loss from the samples was recorded, and the moisture content of each sample was calculated on a wet basis.

#### 2.2.3 Color Analysis

L* (brightness), a* (red/green ratio), and b* (yellow/blue ratio) values of the Turkish delights were measured with a benchtop spectrophotometer (Datacolor 110, Lawrenceville, NJ, U.S.A.). The sample that did not contain corn syrup (SUC) was selected as the reference. Total color change (Δ*E*) was calculated as follows:
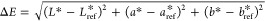
1

#### 2.2.4 Texture Profile Analysis (TPA)

The TPA test was performed using a texture analyzer (Brookfield Ametek CT3, TA44 probe, Middleboro, MA, U.S.A.) by following the method of Delgado and Bañón,^33^ with some modifications. The samples were compressed twice with a cylindrical probe (4 mm in diameter). The testing conditions were 2 consecutive cycles of 50% deformation, crosshead moved at a constant speed of 1 mm/s, and a trigger point of 0.05 N.^33^ Hardness, adhesiveness, cohesiveness, springiness, gumminess, and chewiness values of the Turkish delights were calculated using TPA curves.

#### 2.2.5 X-ray Diffraction

X-ray diffraction experiments were conducted using a Rigaku Ultima-IV X-ray diffractometer (Japan) at 40 kV and 30 mA. Data were collected by the method of Ilhan et al.^20^ between 4° and 70° with a scan rate of 1°/min.

#### 2.2.6 TD NMR Relaxometry Experiments

TD NMR relaxometry measurements were conducted using a 0.5 T (20.34 MHz) NMR instrument (Spin Track, Resonance Systems GmbH, Kirchheim/Teck, Germany). *T*_1_ (spin–lattice) and *T*_2_ (spin–spin) relaxation times were measured for different formulations. For *T*_1_ measurements, the saturation recovery sequence was used with a 300 ms relaxation period (TR), 300 ms observation time, and 4 scans. For *T*_2_ measurements, the Carr–Purcell–Meiboom–Gill (CPMG) sequence was used with parameters of 100 μs echo time, 128 echoes, and 4 scans.

The *T*_1_ and *T*_2_ data were analyzed by two different approaches, as indicated in the study of Pocan et al.^1^ First, mono-exponential fitting was conducted on the relaxation curves using MATLAB. Non-negative least square analysis was also conducted on *T*_2_ curves to obtain a relaxation spectrum. Relative areas (RAs, %), number, and amplitudes of peaks of the samples were recorded using this method with XPFit (Softonics, Inc., Israel). For the *T*_1_ relaxation time, only the mono-exponential approach was used.

#### 2.2.7 FFC NMR Relaxometry Experiments

FFC NMR measurements were performed, and proton *T*_1_ (spin–lattice) relaxation times were measured over different ranges of magnetic field strengths (covering the Larmor frequencies from 10 kHz to 20 MHz) by a FFC NMR relaxometer (Spinmaster FFC2000, Stelar s.r.l., Mede, Italy) to detect the differences in NMRD profiles of the samples prepared with different sugar types. In the proton Larmor frequency range below 10 MHz, the pre-polarized (PP) sequence with the polarizing magnetic field corresponding to 20 MHz was applied for a time of 5*T*_1_, whereas for experimental conditions above 10 MHz, the non-polarized (NP) sequence was used. As a result of a mono-exponential decay/recovery of the amplitude of magnetization versus time (including 22 logarithmically scaled points), the single *T*_1_ relaxation times were calculated for each sample under investigation. The error of the relaxation rates (*R*_1_ ≡ 1/*T*_1_) does not exceed 5%. The NMR signal measured in the samples by FFC came only from the “mobile” protons associated mainly with water molecules undergoing different molecular dynamics depending upon local surroundings. The “rigid” protons associated with a part of the gel candy composition that are not exposed to water were undetectable under the applied measuring conditions because of the short free induction decay (FID) signal. The FFC NMR experiments were conducted at 2 different temperatures, i.e., at room temperature (25 °C) to simulate storage conditions and additionally at 4 °C to see the effect of the temperature on molecular dynamics and confirm the theoretical model applied for analysis. All samples were cooled using a liquid nitrogen (LN_2_) Dewar, and the temperature was stabilized within ±0.1 °C. Additional *T*_1_ measurements at 500 MHz were performed with a Bruker Avance III HD spectrometer coupled to a superconducting Ascend magnet operating at 11.74 T. *T*_1_ relaxation times were determined by the zero method [*t*(*M*_*z*_ = 0) = *T*_1_ ln 2]. OriginPro software with implemented functions was used for fitting theoretical models to NMRD experimental data.

#### 2.2.8 Statistical Analysis

All measurements were carried out in replicates (two and three depending upon the measurement) and reported as means and standard errors. Statistical analysis for all of the experimental results was performed by analysis of variance with the one-way model tool of Minitab (Minitab, Inc., Coventry, U.K.). For the comparison of results, Tukey’s comparison test was applied at a 95% confidence interval. The correlation coefficients were also expressed by Pearson correlation at a 95% confidence level.

## 3 Results and Discussion

### 3.1 Moisture Content

**Figure 1:**
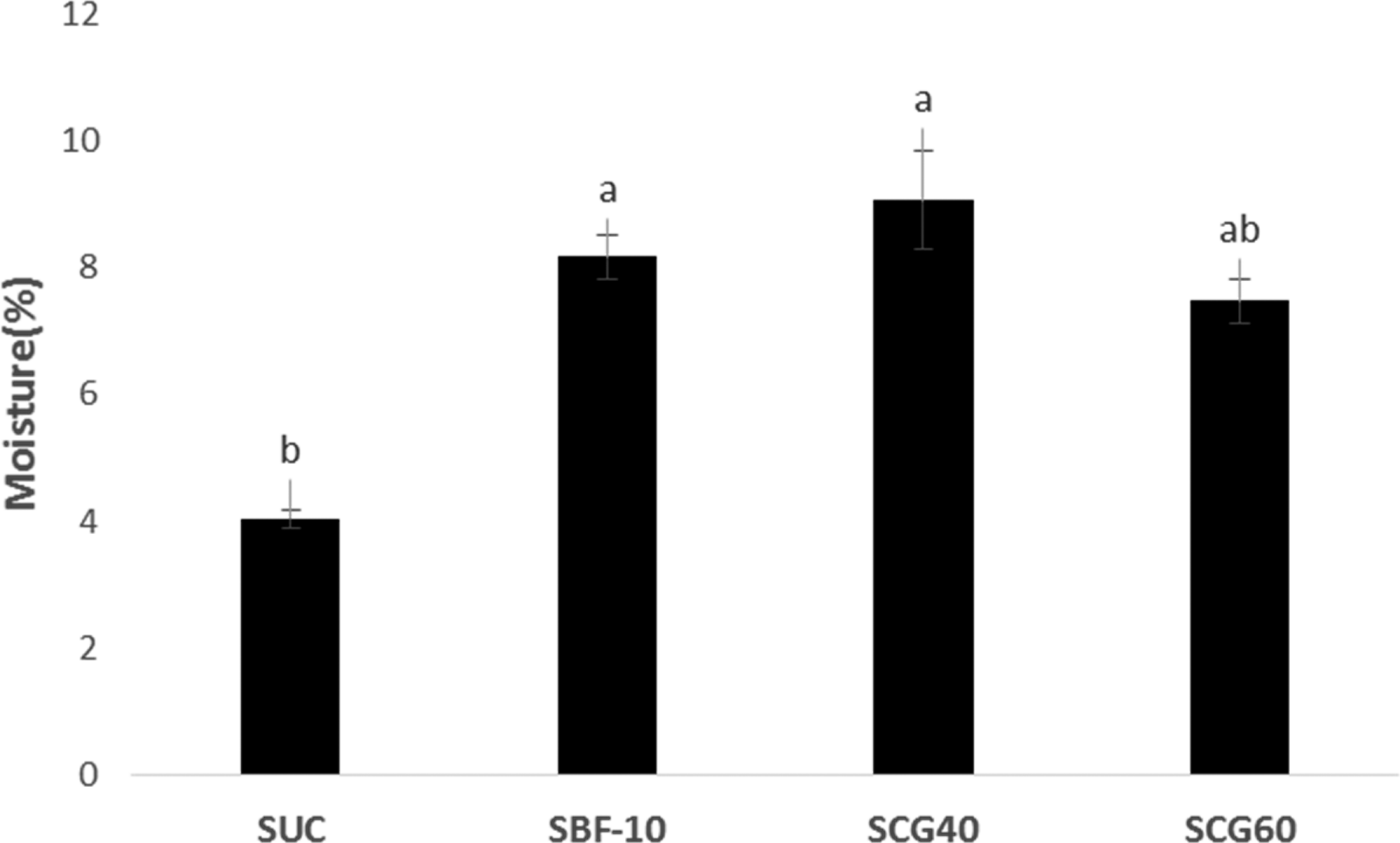
Moisture contents (%) of Turkish delights formulated with different types of sugar source (corn syrup or sucrose). Data were recorded with standard errors. Lowercase letters denote significant difference between the samples at the 95% confidence level between the parameters. Analysis was performed on the basis of two replicates.

The moisture content is one of the most important criteria for the confectionery products because it directly affects the textural and sensorial properties of the products, leading to changes in their shelf life.^1^ This case is also valid for the Turkish delights, which can be classified as a traditional confectionery product. As shown in Figure 1, utilization of corn syrup led to significant changes of the moisture content of the original (SUC) Turkish delights (*p* < 0.05). The lowest moisture content (4%) was found for the original Turkish delights containing only powder sucrose as the sugar source. However, when the corn syrup types were used instead of sucrose, a dramatic increase in the moisture content was observed in comparison to the original samples. It was worth mentioning that use of different types of corn syrups (SBF10, SCG40, and SCG60) led to similar changes in the moisture content of the products (*p* > 0.05), and it was found in the range of 7.5–9%. It was an expected trend because it was a very well-known fact that candies produced using corn syrup can readily pick up moisture as a result of their hygroscopic (water binding) nature.^34^ Hygroscopic substances are also known as humectants, and they promote the retention of water and are capable of keeping the confections moist.^34^ Ergun et al. stated that humectants can be considered as molecules containing hydroxyl groups, which have an affinity to form hydrogen bonds with the molecules of water. Actually, these interactions related with “hydration” occur for all types of sugar,^35^ but for the corn syrups especially with a high dextrose equivalent (DE) value (e.g., SCG40 and SCG60) and containing fructose (e.g., SBF10), they occur to a higher extent compared to sucrose.^34^ Especially for our case, because SBF10, SCG40, and SCG60 samples were produced using only corn syrup as the sugar source, a higher moisture content of these samples is not a surprising outcome.

In addition, as indicated in the Introduction, corn syrups are generally preferred by the manufacturers as a result of their crystallization-inhibiting properties, which is a desirable case for the confectionery gels.^5^ Therefore, confectionery products generally consist of more glucose syrup than sucrose.^36^ Unlike the crystallization inhibition feature of corn syrup, confectionery gels containing sucrose generally have a higher crystallinity degree and lower moisture content,^1^ which is consistent with the fact that crystal structures hold less water.^6^ For example, sucrose can be considered as one of the pure crystalline ingredients, and for these substances, water is only able to interact by hydrogen bonding at the surface of the crystal structure because of the packing arrangement of the crystal lattice, excluding foreign molecules, like water.^34^ Therefore, the lower moisture content of the original sample (SUC) could be attributed to sucrose, which is prone to more crystallization compared to its counterparts containing corn syrups. Moreover, for these samples (SUC), a sandy appearance, which can be considered as a poor quality indicator, was observed most likely as a result of the higher crystallization tendency of sucrose. On the other hand, for its counterparts containing different types of corn syrup, this sandy appearance was not observed.

### 3.2 Color Analysis

The color is an important sensorial and physical property, which affects the perceived quality of the products directly.^4^ It is an also important quality parameter for the Turkish delights. Color analysis was performed for all samples, and L*, a*, b*, and Δ*E* values were reported, as shown in Table 3. SBF10 samples had the highest L* value, meaning that they are the lightest samples (*p* < 0.05), while SCG40 samples were the darkest samples. Similar to the lightness (L*) values, the highest a* (refers to redness) and b* (refers to yellowness) values were also found for the SBF10 samples (*p* < 0.05), meaning that redness and yellowness predominated for these samples. As expected, total color change (Δ*E*) was also significantly higher for SBF10 compared to its counterparts, indicating that this sample was browner than the others (*p* < 0.05). This intense color of the SBF10 sample could be attributed to the enhanced rate of the caramelization reaction. Because Turkish delights used in this study were prepared at 125 °C and it was known that caramelization reactions likely occur above 120 °C,^37^ this was an expected result. According to the study of Kocadaǧlı and Gökmen,^37^ it was known that contribution of fructose to browning development is generally higher than glucose during the caramelization reactions. Therefore, considering that SBF10 is the only sample that contains fructose (10%), a browner color of this sample is actually not surprising. Although the SCG60 sample is not as brown as the SBF10 sample, it was worth mentioning that its total color change (Δ*E*) was higher than that of the SCG40 sample. It was also an expected result because SCG60 contains a higher amount of glucose (60%) compared to the SCG40 sample (40%). Although glucose is not as reactive as fructose during caramelization reactions, it also takes place in caramelization reactions as a reactant. Therefore, a relatively higher amount of glucose (60%) found in the SCG60 sample might have contributed to the formation of the browner appearance compared to the SCG40 sample consisting of 40% glucose.

**Table 3:** L*, a*, and b* Values of Turkish Delights Formulated with Different Types of Sugar Source (Corn Syrup or Sucrose)^a^

sample	L* value	a* value	b* value	Δ*E*
SUC	30.98 c	0.18 c	1.05 b	
SBF10	37.35 a	0.38 a	1.29 a	6.38 a
SCG40	30.20 d	0.14 d	0.38 d	1.03 c
SCG60	33.67 b	0.25 b	0.65 c	2.72 b

a Data were recorded with standard errors. Lowercase letters denote significant difference between the samples at the 95% confidence level between the parameters. Analysis was performed on the basis of two replicates.

Another important point that should be mentioned here is that relatively higher a* and b* values were observed for the original samples (SUC) compared to SCG40 and SCG60 samples. This was an unexpected result because this sample contains only sucrose as the sugar source. At this point, the hypothesis that comes to mind is the “inversion reaction”. It was a very well-known fact that, in the presence of acid, sucrose degrades into fructose and glucose with the help of a high cook temperature and low pH.^38^ Sucrose inversion is also an important reaction that should be considered during the production of lokum (Turkish delights) because acidification is generally used to improve the quality, texture, and flavor of the Turkish delights.^39^ In this study, Turkish delights were also produced using citric acid (0.1%). Therefore, inversion of sucrose to glucose and fructose is an expected result, leading to the formation of a yellowish and reddish color of this sample compared to samples containing only glucose syrup (SCG40 and SCG60) because fructose is a more reactive sugar than glucose in caramelization reactions, as mentioned previously.^37^

Considering the quality of Turkish delights in terms of color parameters, one can conclude that SBF10 samples composed of corn syrup consisting of 10% fructose and 36% glucose were samples with higher quality because they have the lightest samples, which is an important quality indicator for Turkish delights.^40^

### 3.3 Texture Profile Analysis (TPA)

**Figure 2:**
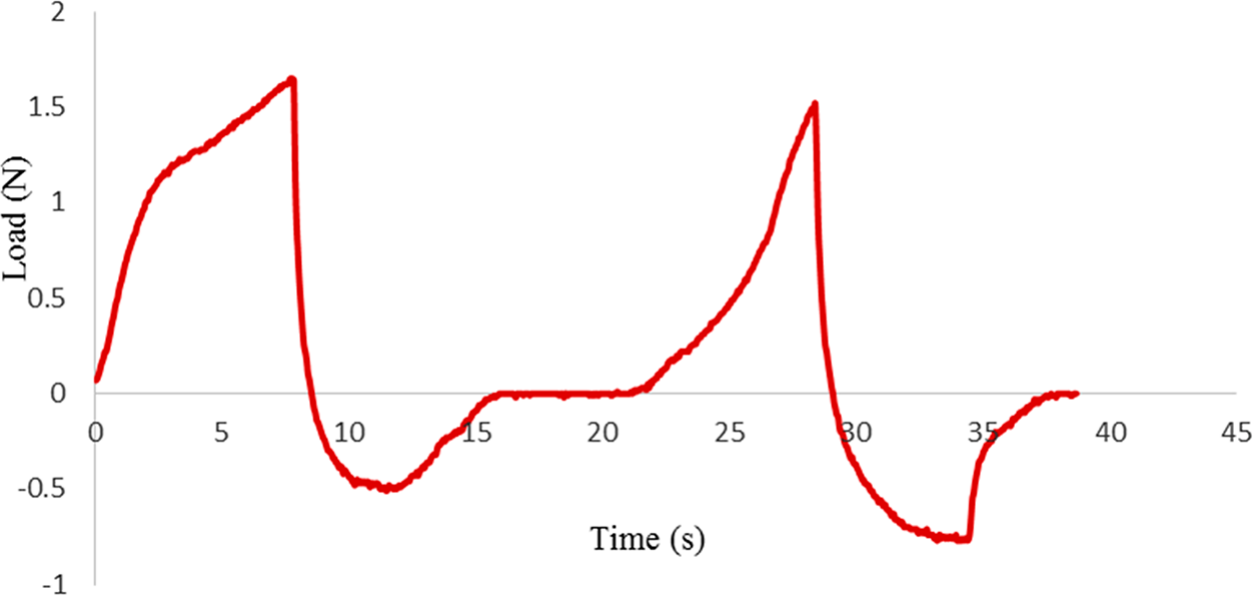
Representative TPA curve for the SBF10 sample.

In addition to the color analysis, TPA also gives valuable information about the quality of confectionery gels. Therefore, TPA was performed for the Turkish delights composed of different types of sugar source, and the results were represented in Table 4. All textural parameters (hardness, adhesiveness, cohesiveness, etc.) were calculated considering the TPA curve (Figure 2).

**Table 4:** Hardness, Adhesiveness, Cohesiveness, Springiness, Gumminess, and Chewiness Values of Turkish Delights Formulated with Different Types of Sugar Source (Corn Syrup or Sucrose)

sample	hardness (N)	adhesiveness (g cm)	cohesiveness	springiness (mm)	gumminess (N)	chewiness (g cm)
SUC	0.64 c		0.19 c	2.08 ± 0.04 c	0.13 ± 0.04 d	
SBF10	1.78 ± 0.07 b	23.50 ± 0.29 a	0.43 ± 0.02 a	6.36 ± 0.05 a	0.76 b	49.00 ± 0.58 a
SCG40	2.60 ± 0.03 a	11.00 ± 0.58 b	0.37 ± 0.01 ab	5.08 ± 0.19 ab	0.95 ± 0.03 a	49.00 ± 2.89 a
SCG60	1.92 ± 0.05 b	5.50 ± 0.29 c	0.28 ± 0.02 bc	3.74 ± 0.40 bc	0.53 ± 0.02 c	20.00 ± 2.89 b

The hardness is an important textural parameter, which is related to the strength of the gel structure under compression, and it is defined as the peak force during the first compression cycle.^41^ As seen in Table 4, the highest hardness value (2.60 N) was found for the SCG40 sample, whereas the lowest hardness was found for the original (SUC) sample (*p* < 0.05). With regard to the desirable textural properties, confectionery gels should not be too hard or too soft.^21^ This case is also valid for the Turkish delights, and consumers usually do not prefer too rough of products.^4^ In this context, it was worth mentioning that such a low hardness value of SUC samples is not a desirable textural property and might be considered as an indication of weak gel formation. A decrease in hardness values of starch-based confectionery gels is generally associated with the phase separation that occurred as a result of the release of water from the gel network, resulting in softening of the sample.^20^

Adhesiveness is another textural parameter, and it is defined as the capacity of a material to stick to another substance; therefore, it depends upon the surface characteristics of the material.^42^ It can be calculated as the negative area between two compression cycles.^42^ It is usually considered as an undesirable characteristic for the confectionery gels because it is related to the stickiness of the food materials.^33^ For the SUC sample, the adhesiveness value could not be reported because the negative area could not be observed in its TPA curve. On the other hand, the highest adhesiveness value was found for the SBF10 samples, whereas the lowest value was found for the SCG60 sample (*p* < 0.05). This outcome might stem from the different stickiness behaviors of corn syrups used in this study. In a previous study, the effect of saccharide distribution on the stickiness of various types of syrups was studied, and it was revealed that allulose syrups had higher stickiness compared to other common types of corn syrups.^43^ Remembering that allulose is a C-3 epimer of fructose, having very similar properties to it, the highest adhesiveness value for SBF10, which was the only sample containing fructose (10%), was not surprising.

The cohesiveness is also known as consistency, and it indicates the strength of internal bonds that make up the body of food and the degree to which a food can be deformed before it breaks.^41^ As indicated by Chandra et al., it was also defined as the ratio of the positive force area during the second compression to that of the first compression observed in the TPA curve. Because cohesiveness indicates the ability of the food to hold together,^41^ higher cohesiveness values could be considered as the formation of a strong gel network that resists rupturing. Referring back to the results that were shown in Table 4, the cohesiveness values of lokum samples were found in the range of 0.19–0.43. Cohesiveness of the SBF10 sample was found to be higher than others, while the smallest cohesiveness was found for the SUC samples. This outcome is consistent with the hardness results that were mentioned above, and low cohesiveness could also be considered as an indication of weak gel formation.

The springiness is another textural parameter, which is related to the elasticity of the sample. Springiness in TPA is related to the height that the food recovers during the time that elapses during the end of first bite and the start of the second bite.^41^ Higher springiness values were obtained for the SBF10 and SCG40 samples, indicating enhanced elastic properties of these products, while the lowest elasticity was obtained for the SUC samples.

As indicated by Delgado and Bañón,^33^ gumminess and chewiness are generally used as the texture descriptors particularly applicable to jelly confections together with hardness. Gumminess is defined as the product of hardness and cohesiveness; therefore, higher hardness led to high gumminess in confectionery gels, and it is considered as an important textural parameter for semi-solid foods.^41^ In our study, the highest gumminess was found for the SCG40 samples, while the lowest gumminess was found for the SUC samples (*p* < 0.05). Because the same trend was also observed for the hardness results, the expected trend was seen in the gumminess of the Turkish delight samples.

The last textural parameter seen in Table 4 was the chewiness, and it was defined as the measure of energy that is necessary to masticate the food and is generally reported for solid foods.^41^ It is calculated as the product of gumminess and springiness that is equal to hardness × cohesiveness × springiness.^41^ As seen in Table 4, the highest chewiness values were found for the SBF10 and SCG40 samples (49.00 g cm).

To conclude, with regard to the TPA of the samples, it is definite that the textural property of Turkish delights follows this order: SBF10 ≥ SCG40 > SCG60 > SUC. According to this trend, it was worth mentioning that utilization of corn syrups, especially SBF10 and SCG40, led to the formation of desirable textural characteristics in Turkish delights. In previous studies, it was also revealed that higher values of textural properties, except adhesiveness, were found to be an indicator of the production of Turkish delights with enhanced textural properties, which was also found parallel to the sensory analysis results.^44^ However, it should be kept in mind that, although utilization of corn syrup in Turkish delights led to the formation of high-quality products with enhanced textural properties, it will also affect the authenticity of this traditional confectionery negatively.

### 3.4 X-ray Diffraction Analysis

**Figure 3:**
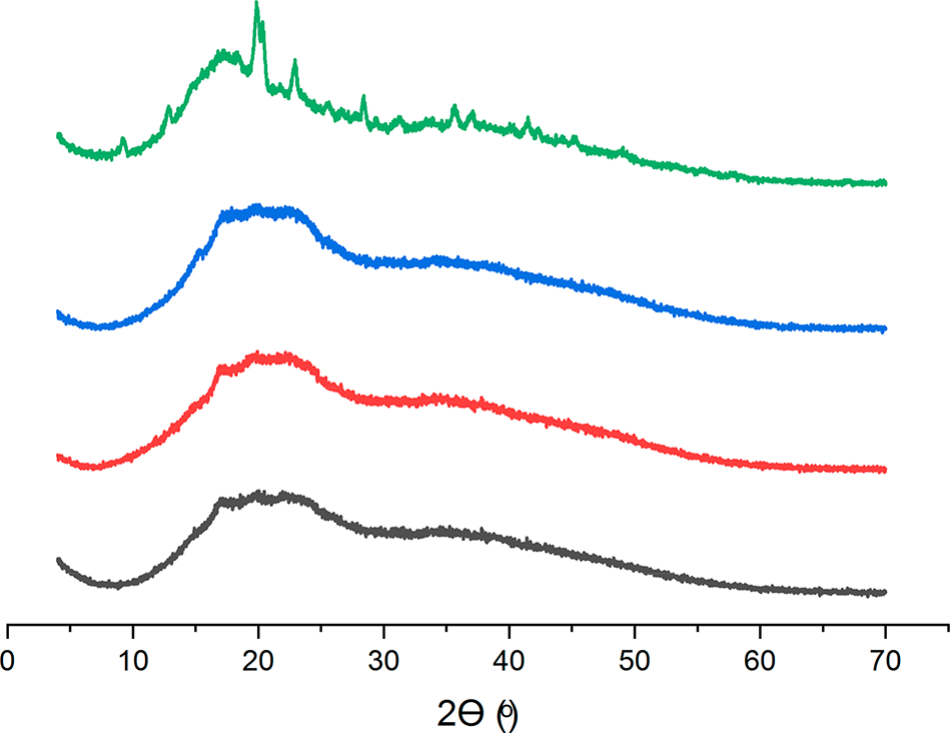
X-ray diffraction pattern of Turkish delights formulated with different types of sugar source (SUC, green; SCG60, blue; SCG40, red; and SBF10, black).

X-ray diffraction analysis of Turkish delights was performed, and patterns of the samples are obtained, as seen in Figure 3. While interpreting the X-ray diffraction pattern, it is important to keep in mind that the narrower and more concentrated peaks are associated with the crystal regions, whereas the larger and less dense peaks are associated with the amorphous regions.^20^ In that regard, it is obvious that SUC samples are the samples with the highest crystallinity degree compared to the corn-syrup-containing counterparts, by demonstrating various sharper and narrower peaks in the X-ray pattern. This case is an expected outcome because corn syrups have a crystallization inhibition nature as mentioned previously, and that is why manufacturers prefer to use corn syrups in the production of Turkish delights, even if this jeopardizes the originality of the products. On the other hand, the X-ray pattern of the corn-syrup-containing samples (SBF10, SCG40, and SCG60) indicated less crystallinity because they demonstrated broader peaks, which distributed in a wide-angle range, and they all showed similar patterns. From previous studies, it was known that starch gives diffraction peaks in the range of 15–24°.^45^ These peaks were also observed for all samples in our study, and the results are not surprising because all samples that were used in our study contain starch as the gelling agent. In addition to these peaks, SUC samples also demonstrated the characteristic diffraction pattern of the sucrose crystal (11.6–24.6°) band.^1^

With reference back to the diffraction pattern of the corn-syrup-containing samples, the amorphous halo pattern is more dominant, as seen in Figure 3. This incident might stem from the existence of maltose in the formulation of corn syrups, found normally in the amorphous state in its native form.^46^ Although similar diffraction patterns were obtained for the corn-syrup-containing samples, there were also small changes. For example, the diffraction pattern of SCG40 and SCG60 samples is very similar, as expected, because these specimens are composed of a similar corn syrup. The only difference is that SCFG40 contains 40% glucose, while SCG60 contains 60% glucose. However, it should be considered that SCG40 contains a higher amount of maltose compared to SCG60 because a small amount of maltose was converted to glucose (40%) during the production of corn syrup, which the SCG40 sample contains. This case also affected the diffraction pattern of these samples. As indicated by Wu et al., native maltose gives a peak at 12.7°.^46^ This peak was also observed in our study for all of the samples that contain corn syrup as a result of the existence of maltose residues. However, an important result that should be mentioned at this point is the different intensities of this peak that were seen among SCG40 and SCG60. The aforementioned peak intensity was found to be higher for the SCG40 sample compared to the SCG60 sample. This case could be related to the quantity of the maltose crystal, as mentioned in a previous study.^46^ According to this study, an increase in the quantity of maltose crystals resulted in an increase in the intensity of related peaks. A similar case might be valid for our study. Because the SCG40 sample includes a higher amount of maltose, the existence of a higher amount of maltose crystals is also possible for this sample, indicating that it has a more crystalline and ordered structure compared to its corn-syrup-containing counterparts.

For the SBF10 sample, all peaks were found to have less intensity compared to the counterparts, indicating that SBF10 had the least crystal structure. It was an expected trend because, as indicated by Pocan et al.,^1^ allulose (which is a C-3 epimer of fructose and shows very similar properties to it) was found to have a crystallization inhibition effect on gelatin-based soft candies. Therefore, a similar effect might be also valid in our study, and even a low amount of fructose that was found in the SBF10 sample might have led to the formation of less crystals.

Consequently, it is worth noting that sucrose-containing original samples had the highest crystallinity, while corn-syrup-containing adulterated samples have a lower crystallinity degree. Results also revealed that adulterated samples could be easily discriminated from the original samples with the help of X-ray diffraction analysis.

### 3.5 *T*_2_ (Spin–Spin) Relaxation Spectra

With regard to the multi-compartment of gel systems, including soft candies, a multi-exponential approach was generally used for interpreting *T*_2_ (transverse relaxation) times.^1^ With the help of inverse Laplace transformations, the decaying magnetization curve could be converted into a continuous one-dimensional distribution of transverse magnetization, resulting in obtaining *T*_2_ relaxation spectra.^1^ A multi-exponential approach was used in various studies related to soft candy products previously, such as gelatin,^47^ starch,^20^ and pectin^21^ based soft candies, concluding that a bi-exponential model is better compared to a mono-exponential model for comparing *T*_1_ relaxation times.

In our study, with the help of XPFit software, discrete component analysis of decaying *T*_2_ curves was performed and two distinct peaks (P1 and P2) with different relaxation times (*T*_2a_ and *T*_2b_) and different relative areas (RAs) were found for all samples, as seen in Tables 5 and 6. The RAs are calculated regarding the magnitude of signal intensity, which was related to each proton pool, and they showed the contribution of these proton pools to the whole signal.^1^

**Table 5:** Proton Spin–Spin Relaxation (*T*_2_) Times (ms) of Each Compartment Observed in the Relaxation Spectrum for Turkish Delights Formulated with Different Types of Sugar Source (Corn Syrup or Sucrose)^a^

sample	*T*_2a_ (ms)	*T*_2b_ (ms)
SUC	2.31 ± 0.04 a	13.17 ± 0.18 a
SBF10	1.14 ± 0.05 b	7.38 ± 0.19 b
SCG40	0.51 ± 0.01 c	5.59 ± 0.11 c
SCG60	0.58 ± 0.01 c	5.17 ± 0.12 c

a Data were recorded with standard errors. Lowercase letters denote significant difference between the samples at the 95% confidence level between the parameters. Analysis was performed on the basis of two replicates.

**Table 6:** Relative Area (RA, %) of Each Peak Observed in the Relaxation Spectrum for Turkish Delights Formulated with Different Types of Sugar Source (Corn Syrup or Sucrose)^a^

sample	RA1 (%)	RA2 (%)
SUC	54.5 ± 1.06 d	45.5 ± 1.06 a
SBF10	59.5 ± 0.35 c	40.5 ± 0.35 b
SCG40	67.5 ± 0.35 b	32.5 ± 0.35 c
SCG60	74.5 ± 0.35 a	25.5 ± 0.35 d

a Data were recorded with standard errors. Lowercase letters denote significance difference between the samples at the 95% confidence level between the parameters. Analysis was performed on the basis of two replicates.

As in the case of previous studies, P1 was generally associated with the non-exchanging proton pool^47^ and was attributed to the rigid proton interactions, which were not exposed to water,^1^ while P2 was thought to be associated with relatively more mobile water, which was entrapped in the gel network.^47^ Therefore, RA1 (%) indicates the contribution of the non-exchanging proton pool, while RA2 (%) shows the contribution of the signal coming from more mobile water entrapped in the gel network to the whole signal.

Compartments with the lowest relaxation times were generally associated with solid–solid interactions,^20^ which might stem from sugar–starch or sugar–sugar interactions in our case. As seen in Table 5, use of corn syrups in the formulation of Turkish delights led to a significant decrease in *T*_2_ relaxation times of P1 compared to the original SUC sample (*p* < 0.05). On the other hand, similar *T*_2a_ relaxation times were found for the SCG40 and SCG60 samples. With regard to RA1 (%) of the samples, a detectable increase was observed for the corn-syrup-containing samples, and all RA1 results were found to be significantly different (*p* < 0.05). The ascending trend of RA1 (%) is actually not surprising because it indicates the enhanced solid–solid interactions, which are expected for the corn-syrup-containing samples because they include various types of solutes, such as maltose, oligosaccharides, etc., in addition to sugar. Contrary to RA1, the descending trend of *T*_2a_ was also expected because more solid existing results in a competitive environment for water, leading to a decrease in *T*_2a_, as in the case of a similar study.^20^ At this point, it was also worth mentioning that a significant high correlation (*r* = −0.94) was found between the hardness values and *T*_2a_ relaxation times of the samples, indicating that, as the solid–solid interactions increase, hardness of the samples decreases. An increased hardness of corn-syrup-containing samples was also mentioned previously in the Texture Profile Analysis (TPA) section. Therefore, it could be concluded that enhanced solid–solid interactions led to an increase in hardness values of samples, indicating the formation of a strong gel formation, which is validated by *T*_2a_ relaxation times.

As mentioned previously, the second compartment (P2) was attributed to water having a higher mobility that was entrapped in the gel network. A similar decreasing trend was also found for *T*_2b_ as in the case of *T*_2a_, and the shortest *T*_2b_ relaxation times were found for the SCG40 and SCG60 samples (*p* < 0.05). The decrease in *T*_2b_ relaxation times for the corn-syrup-containing samples could be explained with the hygroscopic (water binder) nature of corn syrups. It could be hypothesized that corn syrups bound more water compared to sucrose, leading to a decrease in mobility of water that was entrapped in the gel network. RA2 also validated this case because it decreased for the corn-syrup-containing samples, revealing that the signal coming from a more mobile water pool decreased for the corn-syrup-containing samples. On the other hand, the highest *T*_2b_ was found for the SUC sample, indicating a weak gel formation, as mentioned in earlier sections. Most likely, the weak gel formation free water fraction in the gel network increased, leading to an increase in *T*_2b_ and RA2 of SUC samples.

In addition to these findings, it is also very important to mention the power of *T*_2_ relaxation spectra to discriminate corn-syrup-containing samples from the original SUC sample. As clearly indicated in Tables 5 and 6, use of different types of corn syrups in Turkish delights gave rise to the shifting of both peaks toward shorter relaxation times and an increase in RA of P1, while a decrease in RA of P2. Similar adulteration detection studies also exist in the literature. Therefore, it could be considered that *T*_2_ relaxation spectra obtained from a low-resolution system could be used as an authenticity and quality detection tool for Turkish delights and spin–lattice relaxation times (*T*_2a_ and *T*_2b_) and a signal contribution of each pool (RA1 and RA2) could be used as a fingerprint to differentiate the samples.

### 3.6 FFC NMR Relaxometry

The measurements of proton *T*_1_ (spin–lattice) relaxation times as a function of the magnetic field strength were performed to give insight for discriminating the Turkish delight samples in relation to dynamic processes undergoing over the molecular scale. Bearing in mind a board range of time scales of molecular motions occurring in gel-based systems, the FFC NMR experimental points obtained in the frequency range from 10 kHz to 20 MHz were additionally completed with the points obtained at 500 MHz. The latter is essential not only as evidence for proper analysis of the NMRD profiles at a high-frequency range but also because of appropriate evaluation of low-frequency components to the overall relaxation.

**Figure 4:**
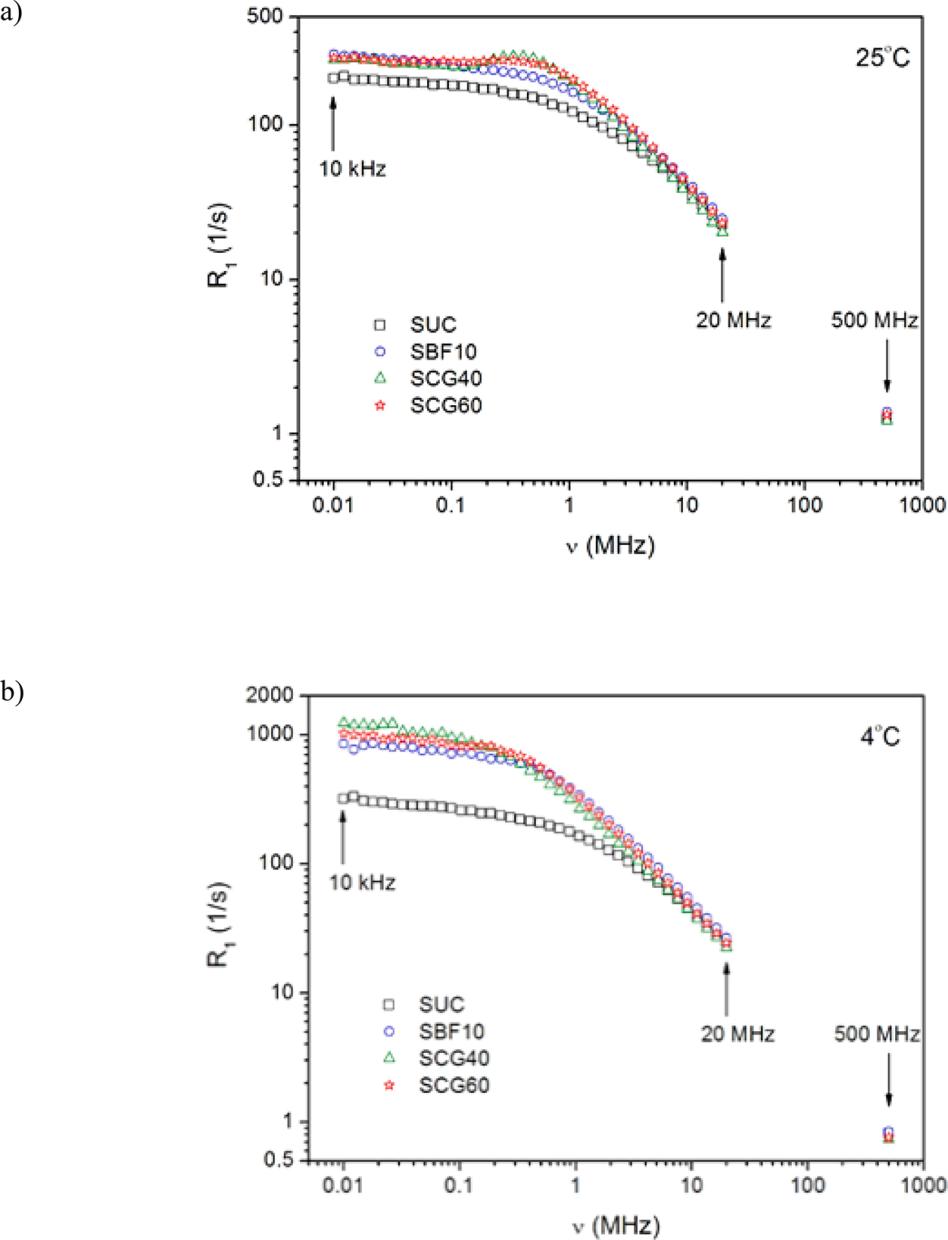
Proton spin–lattice relaxation dispersion profiles of Turkish delights formulated with different types of sugar source (corn syrup or sucrose) obtained with a FFC NMR relaxometer in the range from 10 kHz to 20 MHz at (a) 25 °C and (b) 4 °C. Additional points were obtained at 500 MHz with a conventional NMR spectrometer.

In Figure 4, the experimental spin–lattice relaxation rates (*R*_1_ ≡ 1/*T*_1_) of protons in the samples composed of different types of sugar source are presented as a function of the Larmor frequency (the so-called NMRD profiles) at two different temperatures (25 and 4 °C). As seen, at a low-frequency range (below a few megahertz), the amplitude of the relaxation rate is significantly lower (*T*_1_ relaxation times are longer) in the original SUC sample at 25 °C than that observed in other samples in the same frequency range and temperature (Figure 4a). Differentiation of the *R*_1_ amplitude in the SUC sample and others becomes even more pronounced at 4 °C (Figure 4b). On the other hand, the NMRD profiles recorded for SBF10, SCG40, and SCG60 seem to be similar, except for an enhancement of the relaxation rate observed in the range of 0.15–2 MHz in SCG40 and SCG60 samples at 25 °C. The explanation of this effect requires us to conduct an additional study; therefore, the effect will not be discussed in this paper. However, on the basis of the FFC relaxometry results thus far, we can unambiguously distinguish the original Turkish delight samples (sucrose-containing samples) from the corn-syrup-containing (adulterated) samples. The obtained results indicate that adulterated samples could be easily discriminated from the original samples when conducting even a cursory qualitative analysis of the NMRD profiles recorded at a low-frequency range (below a few megahertz) and at a temperature below the storage temperature for these food products. Ultimately, a single FFC NMR measurement performed under proper conditions, i.e., at a low Larmor frequency (below 1 MHz) and below the storage temperature (e.g., at 4 °C), can be sufficient to confirm with certainty the authenticity of Turkish delights.

To provide a thorough quantitative analysis of the NMRD profiles, a theoretical approach has been carried out related to the molecular dynamics depending upon the microstructure of the food gels. As mentioned previously, different proton fractions can be considered in the systems under investigation. The first fraction of protons (containing “rigid” protons) is associated with the gelator (starch) molecules forming the gel network. These protons, especially protons that are not involved in chemical exchange, are undetectable under the FFC NMR measuring conditions, and thus, they can be neglected for the present study. The second proton fraction (containing “mobile” protons) is associated with mobile molecules of water (as well as mobile sugar molecules). Thinking about a complex microstructure of gel for these molecules and their protons, it is wise to make a distinction into more and less mobile ones depending upon their placement in the local gel structure. The molecules moving within the large pools undergo faster dynamics compared to those entrapped in small pools, where, as a result of the space-confined effect, the molecular dynamics is slower. The described behavior of molecules can apply to both rotational and translational dynamics that modulate intra- and intermolecular dipolar interactions between coupled protons. Consequently, the overall spin–lattice relaxation rate could be expressed by the sum of the individual contributions associated with different proton fractions distinguished in the systems under investigation
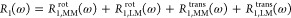
2where MM and LM indices denote more mobile and less mobile fractions, respectively, of protons associated with molecules undergoing rotational (rot) and translational (trans) diffusion and ω (=γ*B*_0_) is the Larmor angular frequency (*B*_0_ is the external magnetic flux density, and γ is the gyromagnetic ratio).

For simplicity and applicability of the above expression to the collected FFC NMR data, the first two terms in eq 2 can be approximated by one rotational contribution reflecting average rotational dynamics. With this assumption, the resulting expression for the proton spin–lattice relaxation rate simplifies to the following form:
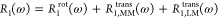
3From a NMR spin–lattice relaxation theory point of view, the rotational and translational dynamics in a different time scale modulate in time dipolar interactions in the spin system; i.e., the former and latter are the main source of fluctuations for the dipolar spin interactions within the same molecules (intramolecular contribution) and between neighboring molecules (intermolecular contribution), respectively. For this reason, two molecular correlation times τ_rot_ and τ_trans_ should be considered in terms of two theoretical models describing rotational and translational contribution in eq 3, respectively.

In simple molecular systems, the model associated with the rotational motion (rotational diffusion or molecular tumbling) is commonly given by a combination of the Lorentzian-shape spectral density, *J*(ω), which is a Fourier transform of the normalized exponential correlation function *G*(*t*) = exp(−*t*/τ_rot_) with a single molecular correlation time τ_rot_^48^
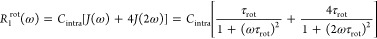
4where *C*_intra_ ∝ 1/⟨*r*⟩^6^ is referred to as the intramolecular dipolar relaxation constant (⟨*r*⟩ denotes the mean distance between coupling proton pairs within the molecule). However, in many complex molecular systems, including molecular gels, a distribution of correlation times is desired. Appling the log-Gaussian distribution form^49^
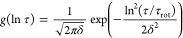
5where τ_rot_ is the correlation time corresponding to the center of the distribution and δ is the width of the distribution, eq 4 can be rewritten as follows:^49,50^
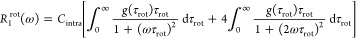
6The form of the *J*(ω) function applying for translational diffusion is dependent upon the model assumed.^48,51^ The one frequently used in viscous liquids is proposed by Torrey.^52^ The contribution to the overall relaxation is given by^52−54^
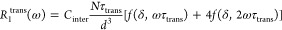
7where *C*_inter_ = (9/8)(μ_0_γ^2^ℏ/(4π))^2^, *d* is the closest distance between the interacting molecules, τ_trans_ is the average time between molecular translational jumps, *N* is the number of protons (spin density) per unit volume, δ = ⟨*a*⟩^2^/(12*d*^2^), with ⟨*a*⟩^2^ = 6*Dτ*_trans_ being the mean square root of the molecular jump distance, *D* is the translational self-diffusion constant, and *f*(δ, *x*) are analytical functions.^52^

**Figure 5:**
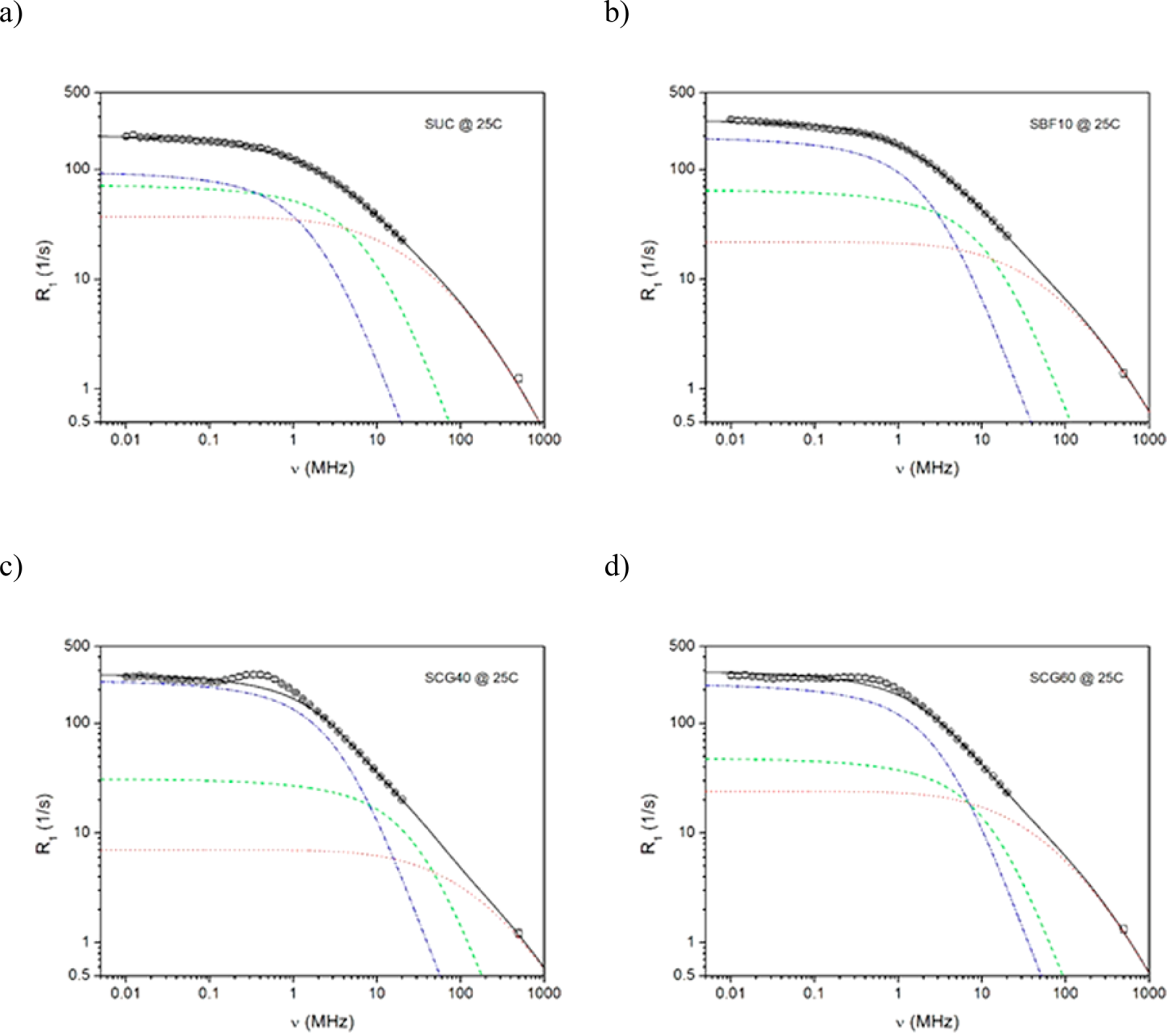
Proton spin–lattice relaxation dispersion profiles obtained at 25 °C for (a) SUC, (b) SBF10, (c) SCG40, and (d) SCG60. The solid lines are the best fits of eqs 3, 6, and 7 to the experimental data (see the text). Deconvolution of the overall fits: (dotted lines) rotational contributions and (dashed and dash-dotted lines) two translational contributions.

**Figure 6:**
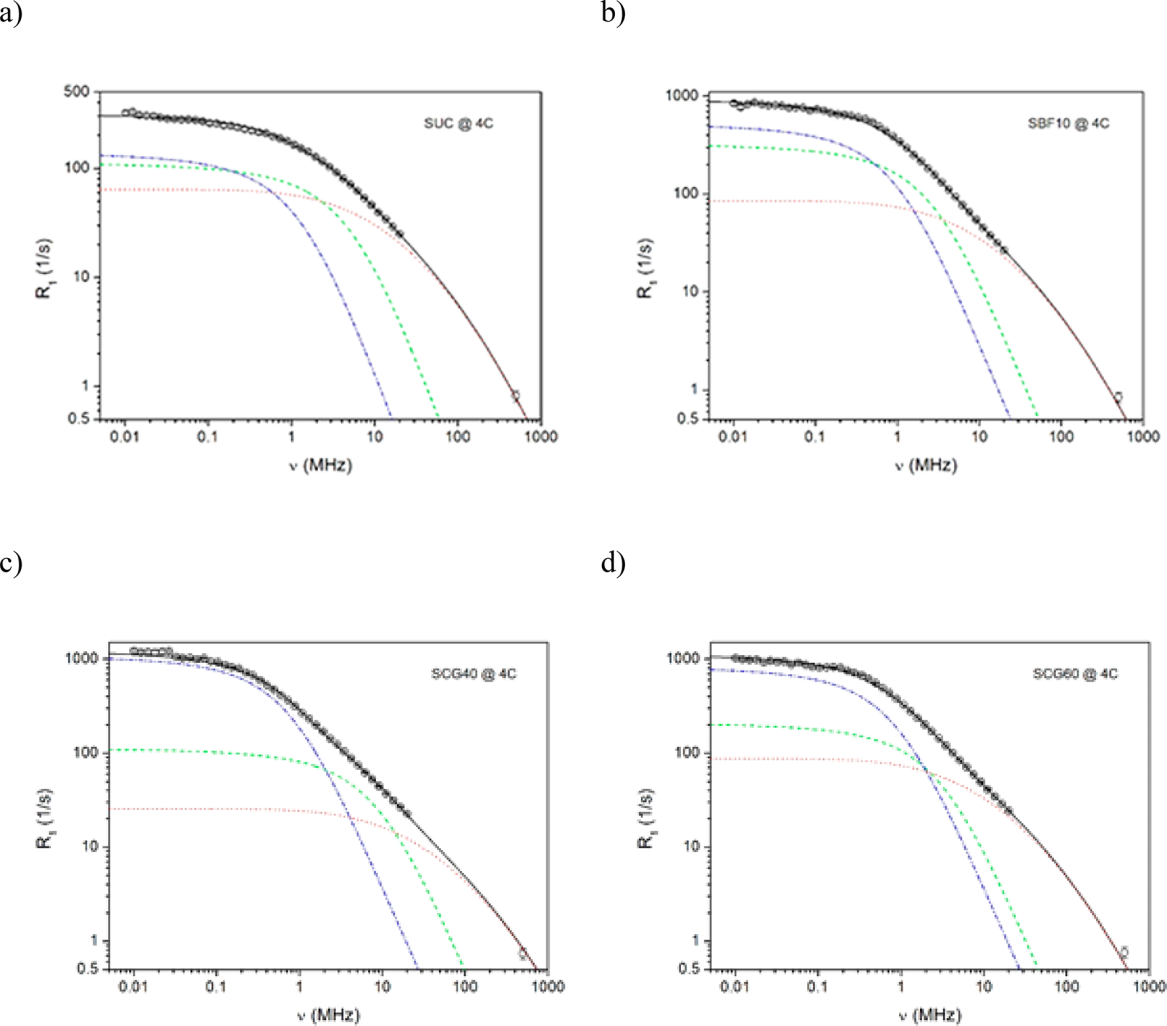
Proton spin–lattice relaxation dispersion profiles obtained at 4 °C for (a) SUC, (b) SBF10, (c) SCG40, and (d) SCG60. The solid lines are the best fits of eqs 3, 6, and 7 to the experimental data (see the text). Deconvolution of the overall fits: (dotted lines) rotational contributions and (dashed and dash-dotted lines) two translational contributions.

The application of the presented theoretical models in combination with eq 3 allowed for the reproduction of the proton NMRD profiles obtained in all studied samples in the broad frequency range of 0.01–500 MHz and determine the molecular parameters characterizing the rotational and translational dynamics of molecules in the gel systems. The results of the conducted analysis are presented in Figures 5 and 6 for the FFC NMR experimental data collected at 25 and 4 °C, respectively. The solid lines are the best fits of eq 3, after insertion of eqs 6 and 7, to the experimental points, whereas the dotted, dashed, and dash-dotted lines represent the individual relaxation contributions associated with rotational (dotted line) and two translational (dashed and dash-dotted lines) motions detected by FFC NMR relaxometry. The multi-parameter fits are satisfactory, and reasonable fitting parameters were obtained, as seen in Table 7. It is worth noting that, for translational contributions, the closest distance *d* between the interacting molecules was kept constant during the fitting procedure. In relation to the diameter of water (2.75 Å) and sugar (∼4.5 Å) molecules, the average value *d* = 3.6 Å was assessed and *a* = *d* (the mean jump length of molecules corresponds to the value *d*) was assumed for simplification. For rotational contributions, the width δ of the log-Gaussian distribution was kept at the level of 1.5 decade. Finally, two parameters for the rotational component (τ_rot_ and *C*_intra_) and two parameters for each translational component (*D* and *N*) were fitted to the experimental data at 4 and 25 °C, whereas spin densities *N*_MM_ and *N*_LM_ for more mobile (MM) and less mobile (LM) fractions, respectively, were fitted only to the experimental data at 25 °C, while for fits at a lower temperature, they were kept as constant parameters.

**Table 7:** Parameters Obtained from the Fits Presented in Eq 7: Intramolecular Dipolar Relaxation Constants (*C*_intra_), Rotational Correlation Times (τ_rot_), Translational Self-Diffusion Coefficients (*D*_MM_ and *D*_LM_) for More Mobile (MM) and Less Mobile (LM) Molecules, and Numbers of Protons Per Unit Volume (*N*_MM_ and *N*_LM_) in Relation to Fractions MM and LM^a^

sample	moisture (%) and relative change	temperature (°C)	*C*_intra_ (×10^9^, s^2^)	τ_rot_ (ns)	*D*_MM_ (×10^–12^, m^2^/s)	*D*_LM_ (×10^–13^, m^2^/s)	*N*_MM_ (×10^28^, m^–3^)	*N*_LM_ (×10^28^, m^–3^)	*N*	relative change in *N*	ratio *N*_MM_/*N*_LM_
SUC	4/–	25	4.20 ± 0.03	0.57 ± 0.06	1.70 ± 0.09	3.90 ± 0.09	4.39 ± 0.07	1.33 ± 0.04	5.72		3.3
		4	3.86 ± 0.07	1.08 ± 0.04	1.11 ± 0.04	2.71 ± 0.08					
SBF10	8.2/2.05	25	4.95 ± 0.05	0.29 ± 0.04	2.83 ± 0.09	5.43 ± 0.06	6.57 ± 0.19	3.79 ± 0.16	10.36	1.81	1.7
		4	3.86 ± 0.04	1.44 ± 0.03	0.58 ± 0.06	2.06 ± 0.04					
SCG40	7.5/1.87	25	4.45 ± 0.11	0.35 ± 0.17	2.67 ± 0.43	6.65 ± 0.12	4.48 ± 0.14	5.37 ± 0.15	9.85	1.72	0.8
		4	3.43 ± 0.08	1.66 ± 0.07	0.62 ± 0.02	1.84 ± 0.03					
SCG60	9.1/2.27	25	4.01 ± 0.09	0.11 ± 0.08	6.51 ± 0.11	7.11 ± 0.07	7.24 ± 0.16	6.2 ± 0.12	13.44	2.35	1.2
		4	3.19 ± 0.03	0.52 ± 0.03	1.83 ± 0.06	1.62 ± 0.02					

a The relative changes in moisture and total *N* (*N* = *N*_MM_ + *N*_LM_) were calculated relative to values taken for the original SUC sample.

The following major conclusions can be made from our FFC NMR data analysis. First, it has not been found before that, in soft candy products, apart from the rotational motions, the molecules (mainly water but also sugar molecules) undergo two types of translational dynamics. The observed two self-diffusion processes are possible to distinguish by FFC NMR relaxometry as a result of significantly different diffusion constants (see Table 7). For instance, for the original Turkish delight sample containing sucrose (SUC), the two diffusion coefficients are of the order of 1.7 × 10^–12^ and 3.9 × 10^–13^ m^2^/s at 25 °C. For this reason, a complex microstructure of these food gels is filled with pools containing more and less mobile molecules, which, as a result of topological limitations of the gel network (confined effect), are not able to average the time scale of two distinguished translational dynamic processes.

The second important fact is that the carried out analysis has provided the spin (proton) densities within the pools with different molecular dynamics. Unexpectedly, the relative change in moisture of SUC, SBF10, SCG40, and SCG60 samples is in good agreement with the relative change in total proton densities (*N* = *N*_MM_ + *N*_LM_), as seen in Table 7. This leads to the conclusions that the observed translational molecular dynamics is mainly determined by water molecules entrapped in the gel network; however, the interactions of water with sugar molecules cannot be neglected. With reference of the ratio *N*_MM_/*N*_LM_ to the number (or even size) of the corresponding proton pools in the studied samples, it is possible to find out the following quantitative correlations: (i) for SUC, almost 3 times more protons are associated with pools containing more mobile molecules, in contrast to these with less mobile molecules; (ii) for SBF10, the ratio *N*_MM_/*N*_LM_ increases to ∼2; and (iii) for SCG40 and SCG60, the proton densities in two considered pools are comparable. Although this analysis requires an additional microstructure study, the results obtained with the use of FFC NMR relaxometry give a direct indication of differentiation between the local microstructure of the studied food gels containing different types of sugar.

To summarize, this study was built on two main purposes. The first purpose is to examine the effect of different types of corn syrup substitution on Turkish delights using important quality parameters, like moisture content, color, crystallinity, and textural parameters (hardness, springiness, adhesiveness, etc.). The second purpose is to discriminate the adulterated (corn syrup)-containing samples from the original samples (sucrose containing) using TD NMR and FFC NMR techniques. Results clearly indicated that corn-syrup-containing samples had improved textural properties and were less prone to crystallization, although this case affected the authenticity of the products negatively. Both TD NMR and FFC NMR techniques were found to be effective to discriminate the original samples from the corn-syrup-containing samples. Thanks to FFC technology, we moved one step further, and quantitative analysis of relaxation behavior of Turkish delights was performed by considering the water dynamics of different proton pools found in samples. Results clearly indicated that, apart from the rotational motions, molecules in Turkish delights (mainly water but also sugar molecules) undergo two types of translational dynamics. In addition, it was demonstrated that translational molecular dynamics is mainly determined by water molecules entrapped in the gel network. This study revealed that both FFC and TD NMR techniques are promising methods, enabling researchers to detect the authenticity and quality of soft candy products, which will pave the way for utilization of low-resolution NMR techniques in the confectionery industry and research and development (R&D) laboratories.
